# Silencing Mutated *β-Catenin* Inhibits Cell Proliferation and Stimulates Apoptosis in the Adrenocortical Cancer Cell Line H295R

**DOI:** 10.1371/journal.pone.0055743

**Published:** 2013-02-07

**Authors:** Sébastien Gaujoux, Constanze Hantel, Pierre Launay, Stéphane Bonnet, Karine Perlemoine, Lucile Lefèvre, Marine Guillaud-Bataille, Felix Beuschlein, Frédérique Tissier, Jérôme Bertherat, Marthe Rizk-Rabin, Bruno Ragazzon

**Affiliations:** 1 Institut Cochin, Université Paris Descartes, CNRS (UMR 8104), Paris, France; 2 Inserm, U1016, Paris, France; 3 AP-HP, Hôpital Cochin, Department of Digestive and Endocrine Surgery, Paris, France; 4 Endocrine Research Unit, Medizinische Klinik and Poliklinik IV, Ludwig-Maximilians-University, Munich, Germany; 5 Rare Adrenal Cancer Network-Corticomédullosurrénale Tumeur Endocrine, Institut National du Cancer, Paris, France; 6 AP-HP, Hôpital Cochin, Department of Pathology, Paris, France; 7 AP-HP, Hôpital Cochin, Department of Endocrinology, Center for Rare Adrenal Diseases, Paris, France; University of Ulm, Germany

## Abstract

**Context:**

Adrenocortical carcinoma (ACC) is a rare and highly aggressive endocrine neoplasm, with limited therapeutic options. Activating β-catenin somatic mutations are found in ACC and have been associated with a poor clinical outcome. In fact, activation of the Wnt/β-catenin signaling pathway seems to play a major role in ACC aggressiveness, and might, thus, represent a promising therapeutic target.

**Objective:**

Similar to patient tumor specimen the H295 cell line derived from an ACC harbors a natural activating β-catenin mutation. We herein assess the *in vitro* and *in vivo* effect of β-catenin inactivation using a doxycyclin (dox) inducible shRNA plasmid in H295R adrenocortical cancer cells line (clone named shβ).

**Results:**

Following dox treatment a profound reduction in *β-catenin* expression was detectable in shβ clones in comparison to control clones (Ctr). Accordingly, we observed a decrease in Wnt/βcatenin-dependent luciferase reporter activity as well as a decreased expression of *AXIN2* representing an endogenous *β-catenin* target gene. Concomitantly, *β-catenin* silencing resulted in a decreased cell proliferation, cell cycle alterations with cell accumulation in the G1 phase and increased apoptosis *in vitro*. *In vivo*, on established tumor xenografts in athymic nude mice, 9 days of *β-catenin* silencing resulted in a significant reduction of *CTNNB1* and *AXIN2* expression. Moreover, continous *β-catenin* silencing, starting 3 days after tumor cell inoculation, was associated with a complete absence of tumor growth in the shβ group while tumors were present in all animals of the control group.

**Conclusion:**

In summary, these experiments provide evidences that Wnt/β-catenin pathway inhibition in ACC is a promising therapeutic target.

## Introduction

Adrenocortical carcinoma (ACC) is a rare and highly aggressive endocrine neoplasm, with a 5-year overall survival of around 40% [1]–[4]. Therapeutic options for these patients are scarce, and available chemotherapies of limited effectiveness. A better understanding of tumor biology and molecular prognostic factors would help to select relevant therapeutic targets and to develop innovative therapeutic strategies.

Activation of the Wnt/β-catenin signaling pathway in adrenocortical tumorigenesis has recently been investigated in detail [5]–[10], and seems to play a major role in adrenocortical carcinoma prognosis. In animal models, a constitutive activation of the Wnt/β-catenin pathway in the adrenal cortex of transgenic mice leads to the development of adrenocortical tumors with malignant characteristics [11]. In humans, this pathway is frequently activated mainly trough β-catenin gene (*CTNNB1*, i.e. Catenin (cadherin-associated protein), beta 1) mutations [5], [7], and is associated with specific clinical and pathological characteristics and a poor outcome [8]. Likewise, a specific transcriptomic signature of tumors with *CTNNB1* mutation has recently been shown [12], and might be responsible for the particular poor prognosis of affected patients. Overall, these observations suggest Wnt/β-catenin signaling pathway inactivation as a promising therapeutic target in ACC.

The aim of this study was to assess the *in vivo* and *in vitro* effects of Wnt/β-catenin signaling pathway specific inactivation using short hairpin RNA (shRNA) in a tumor model for adrenocortical carcinoma (H295R).

## Materials and Methods

### Cell Culture and generation of H295R Clones

Adrenocortical carcinoma cell line H295R stably transfected with the Tet repressor (H295R/TR) was kindly provided by Dr. Lalli [13]. Cells were grown as previously described [14]. pTer-β-catenin vector, which expresses a doxycyclin inducible shRNA targeted *CTNNB1* (*β-catenin*; targeted sequence: 5′-GTGGGTGGTATAGAGGCTC-3′) mRNA, and the control vector (pTer) were obtained from Dr. van de Wetering [15]. H295R/TR were transfected with the pTer-β-catenin or pTer and clones were selected by zeocin (50 µg/ml, InvivoGen). Three shRNA-βcatenin clones (shβ) were selected in which *CTNNB1* expression was down-regulated at least 5 fold in a doxycyclin (dox, 0.2 µg/ml, Sigma) dependent manner in comparison to three control clones (Ctr) transfected with pTer vector. All cell clones were investigated for their ability to express specific steroidogenic genes (*StAR* and *CYP11B1*) and their responses to the cAMP/PKA pathway which was found to be comparable to that of the parental cell line, H295R [16] (data not shown). S45P *CTNNB1* (*β-catenin*) gene activating mutation, previously identified in the parental H295R cell line [5], [8], was confirmed by direct sequencing in all Ctr and shβ clones (data not shown). While data are presented for a single Ctr and shβ clone all *in vitro* experiments were confirmed with equivalent results in 2–3 individual clones (Ctr and shβ).

### Analysis of RNA and protein levels

Total RNA or protein extractions and analysis from cell lines were performed as previously described [14] with primers and antibodies described in Table S1.

### Cell Transfection, and reporter assays

As a Wnt/β-catenin pathway reporter construct driving expression of luciferase gene, the TopFlash plasmid (Top) was used which contains two copies of the β-catenin/T-cell factor TCF-binding sites whereas the FopFlash plasmid (Fop) contains two mutated copies of the β-catenin/TCF-binding sites [5]. Rous sarcoma virus (RSV)-Renilla (Promega) was used as a control of transfection efficiencies. Cells were cotransfected and Firefly and Renilla luciferase activities were sequentially measured as previously described [14].

### Cell proliferation, cell cycle and apoptosis analysis

Proliferation was measured by MTT assay (Promega). The cell cycle and apoptosis were analysed by flow cytometry as previously reported [17].

### Xenograft, pathological examination and immunohistochemical staining

Female athymic NMRI nu/nu mice (6–8 weeks) were purchased from Harlan Winkelmann (Borchen, Germany) and housed under pathogen-free conditions. All experiments were carried out following protocols approved by the Regierung von Oberbayern and in accordance with the german guidelines for animal studies. 15×10^6^ cells of the individual clones were inoculated in a volume of 200 µl PBS subcutaneously into the neck of each mouse. For short-term therapeutic experiments dox treatment was initiated when the longest tumor diameters ranged between 0.2–0.9 cm in size (after 21–31 days). Doxycyline was added in a final concentration of 2 mg/ml to the drinking water in amber water bottles. After 9 days of dox treatment tumors were excised, fixed in formalin, embedded in paraffin, and 4 µm sections cut and stained with Hematoxylin-Eosin-Saffron. Immunohistochemistry for β-catenin was performed as previously described [5]. Cells for long-term therapeutic experiments were inoculated and 3 days after tumor induction mice were starting from then continuously treated with dox water. Tumor size was measured every other day using a calliper as described earlier [18]. At day 31 days after tumor induction, when first tumors reached a longest tumor diameter of 1.5 cm, mice were sacrificed and tumors excised.

### Statistical analysis

All *in vitro* data with statistical analyses represent the quantification of at least three experiments. Control conditions were set as 100% and data were analyzed using Fisher's test. The statistical analysis for comparison of tumor weight after long-term therapeutic experiment *in vivo* was performed by Mann-Whitney test. Significance was set at P<0.05 (represented by * in figures); P<0.01 (**) and P<0.001 (***).

## Results

### Efficient inactivation of *CTNNB1* by shRNA in adrenocortical cancer cells

**Figure 1:**
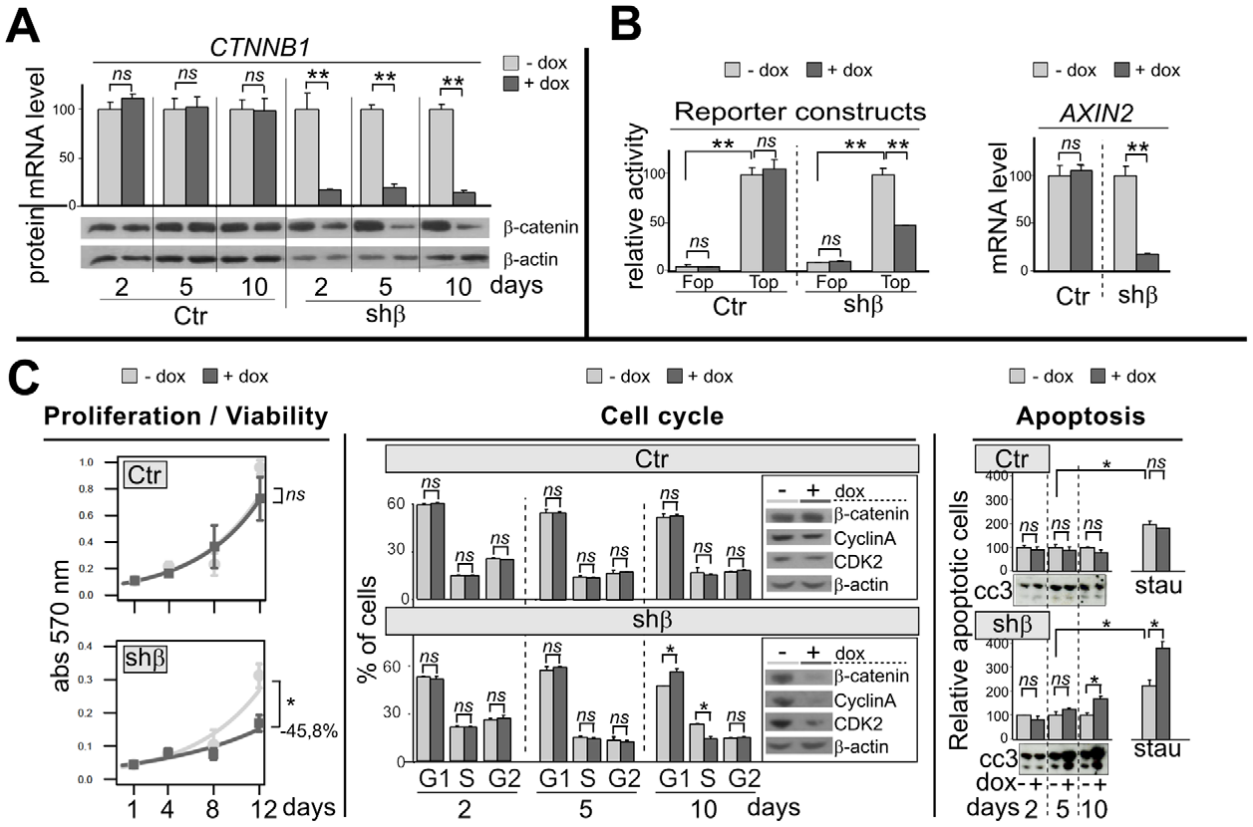
*CTNNB1* silencing alters the Wnt/β-catenin signaling pathway, proliferation, cell cycle and apoptosis. **A**, Histogram and Western blot panels represent *CTNNB1* (*β-catenin*) mRNA and protein accumulation, in Ctr and shβ clones after 2, 5 or 10 days after addition of doxycyclin (dox) in the culture medium (0.2 µg/ml). **B-left**, cells were transiently co-transfected with an artificial Wnt/-β-catenin pathway reporter construc (Top) or his control mutated (Fop). After 24 h, cells were treated by vehicle or dox (0.2 µg/ml) for 24 h and luciferase activity was measured. **-right**, Histogram represent *AXIN2* mRNA accumulation after 2 days of dox treatment. **C**-**left**, Cell survival curve of cells, as assessed by the MTT assay without or with dox (0.2 µg/ml) for 1, 4, 8 or 12 days. **-center**, The distribution of cells in the various phases of the cell cycle was analysed by flow cytometric analysis of propidium iodide staining after vehicle or dox treatment for 2, 5 and 10 days. Western blots show β-catenin, CyclinA and CDK2 protein levels at 10 day. **-right**, Histograms represent apoptotic cells measured by flow cytometric annexin V incorporation, after vehicle or dox treatment for 2, 5 and 10 days, without or with staurosporin co-treatment for last 6 h (0.5 µg/ml). Western blots show the cleaved caspase 3 (cc3) in same condition.

H295R cells, harboring a heterozygous *CTNNB1* gene mutation on the GSK3β phosphorylation site (S45P), exhibit a constitutive transcriptional activity of β-catenin-LEF/TCF [5]. Cell clones expressing a doxycylin inducible shRNA targeting β-catenin were generated. Two days following shRNA-β-catenin induction by doxycyclin (dox) treatment, *CTNNB1* mRNA (−85%; p<0.01) and protein levels were significantly decreased (figure 1-A). This decrease in *CTNNB1* mRNA and protein levels persisted with dox treatment up to 10 days. In contrast, dox treatment had no effect on *CTNNB1* expression in control clone (Ctr) (figure 1-A).

### *CTNNB1* silencing decreases Wnt/β-catenin-LEF/TCF dependent transcription

The Wnt/β-catenin-LEF/TCF dependent transcription was studied by using the Top-Flash/Fop-Flash plasmids (Top and Fop). As expected, transfected H295R cells showed higher transcriptional activity of the β-catenin-LEF/TCF dependent luciferase reporter construct Top in comparison to the mutated Fop construct (figure 1-B, Ctr-Fop: 5% compared to Ctr-Top: 100%, p<0.01; and shβ-Fop: 9% compared to shβ-Top: 100%, p<0.01). H295R cells which expressed shRNA-β-catenin (shβ with dox) showed lower transcriptional activity of the reporter construct Top (shβ-Top: −53%, p<0.01). Dox treatment did not affect activity of the Top construct in the control clone (Ctr-Top) or on the Fop construct with mutated LEF/TCF sites in both clones (Ctr-Fop and shβ-Fop).

Moreover, *CTNNB*1 silencing for two days significantly decreased mRNA level of an endogenous canonical downstream target gene of Wnt/β-catenin pathway, i.e *AXIN2*, in the shβ clone (figure 1-B, shβ: −82%, p<0.01) compared to control clones (Ctr, *ns*).

### *CTNNB1* silencing alters proliferation, cell cycle and apoptosis

A time course study of *CTNNB1* silencing in H295R cell line demonstrated a significant decrease in proliferation (−45,8% at 12 days, p<0.05) compared to shβ clone without *CTNNB1* silencing (−dox) or the control clone (figure 1-C), determined by MTT conversion.

Flow cytometric analysis of cell cycle by propidium iodide staining showed no effect on cell cycle until 5 days. However following 10 days of dox treatment, *CTNNB1* silencing resulted in the accumulation of cells in the G1 phases and a decrease of cell proportion in the S phase (figure 1-C, shβ-10d-G1: 55.3±0.4% *vs* 65.8±2.2%; -S: 27.5±0.2% *vs* 16.5±1.6%, p<0.05). No such difference was observed in the control clone (Ctr). At this time point, we observed a reduction of two important proteins for G1/S transition, Cyclin A and CDK2 only in cells silenced for *CTNNB1* (figure 1-C).

Similarly, no effect on apoptosis assayed by the flow cytometric analysis of annexine V incorporation in cells, was observed until 5 days while 10 days of dox induced *CTNNB1* silencing increased the proportion of apoptotic cells (figure 1-C, shβ-10d: 167%, p<0.05) when compared to cells without dox treatment. No such difference was observed in the control clone. This increase of apoptosis by *CTNNB1* silencing was also confirmed by the gain of a proapoptotic protein level, cleaved caspase3 (cc3), which was detectable already at an earlier time point (5 days, figure 1-C). Likewise, *CTNNB1* silencing increased the apoptotic effect of staurosporin (figure 1-C; shβ-5d+stau: 224% *vs* 377%, p<0.05) while no significant difference was observed in control clones.

### *CTNNB1* silencing abolish xenograft development of ACC cell line

To evaluate the functional significance of β-catenin knock-down on tumor development we proceeded with investigation in a subcutaneous xenograft tumor model in athymic nude mice.

**Figure 2:**
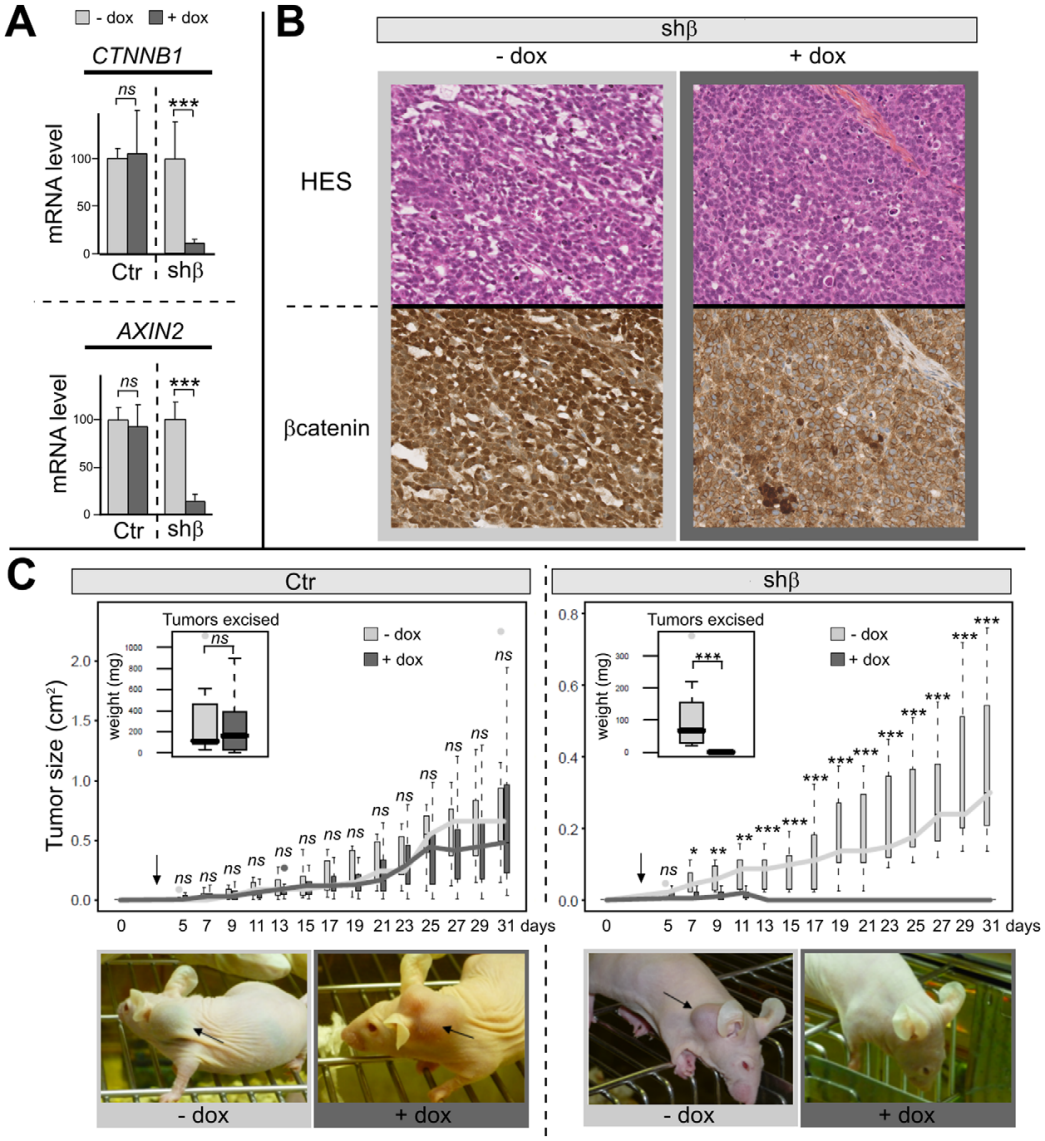
*CTNNB1* silencing abolish xenograft development of ACC cell line. **A**, Histograms represent *CTNNB1* (*β-catenin*) and *AXIN2* mRNA accumulation in xenograft for both Ctr (−dox n = 6; +dox, n = 5) and shβ (−dox, n = 5; +dox, n = 5) clones on established tumors and after 9 days of dox treatment. **B**, hematoxylin–eosin–saffron and β-catenin, staining (×20) on representative tumors of shβ clone without and with dox treatment (same experiment as B). **C**, Boxplots represent the tumor sizes for Ctr and shβ xenografts in mice continuously treated with vehicle or dox after 3 days of tumor induction. Boxplots in the left corner represent the weights of tumors excised. Ctr (−dox n = 7, + dox n = 8), shβ (−dox, n = 7; +dox, n = 8).

In a first step, short term experiments with *CTNNB1* inactivation on established tumors (21 to 31 days after xenografting) for a duration of 9 days were performed to mirror the time course from our *in vitro* experiments (figure 1-C). Similar to the *in vitro* setting mRNA expression analyzes revealed a dox dependent significant decrease in tumoral *CTNNB1* and *AXIN2* expression in shβ clone (figure 2-A; *CTNNB1*: −89%, p = 0.007; *AXIN2*: −87%, p<0.001) while control clones remained unaffected by dox treatment. Moreover, and in accordance with the cell culture experiments, immunohistochemical analysis (figure 2-B) revealed a dox treatment dependent reduction of β-catenin protein. But, we have not observed any differences for the Ki67 and the cleaved caspase3 expression by immunohistochemistry analyzes (data not shown), suggesting that 9 days of dox is not sufficient *in vivo* to induce effects on proliferation and on apoptosis. Because this time course is too short to investigate a potential effect on tumor growth in an *in vivo* model, long term dox treatment was performed. Ctr and shβ clones were subcutaneously injected and 3 days after tumor induction mice were treated with dox in a continuous manner. There is no significant difference after 31 days in tumor size between shβ and Ctr clones in absence of dox treatment, but it's seem that the shβ clone grow slower than the Ctr clone, probably because of a clonal effect. For this reason the doxycyclin-inducible system is suitable, because the clones are their own control. While dox treatment did not affect tumor growth and weight in the control clone (figure 2-C-left, medians weight: 112 mg *vs* 162.7 mg, *ns*), there was a significant impact of *CTNNB1* silencing upon dox treatment in the shβ clone (figure 2-C-rigth). Indeed, for shβ clone, during the first 10 days, tumor growth was observable in both groups (without or with dox) but, after 13 days, no tumor was detectable in any of the mice of shβ group treated with dox, including after dissection and pathological analysis (figure 2-C-rigth, median weight 67.4 mg *vs* 0 mg, p<0.001).

## Discussion

In many cancers, the Wnt/β-catenin signalling pathway plays an important role regulating cell growth, motility, and differentiation [19]. We and others have previously demonstrated the importance of the Wnt/β-catenin signaling pathway activation in adrenal cortex tumorigenesis [5]–[10]. This activation is associated with a specific molecular signature and a worse outcome with lower overall survival [12]. Doghman and colleagues previously showed that a TCF antagonist inhibits proliferation of adrenocortical cells H295R, suggesting a central role of Wnt/β-catenin pathway in adrenocortical tumorigenesis [20]. However, this pharmacological approach might not be specific for Wnt signaling. We herein, using both *in vitro* and *in vivo* experiments, demonstrate that direct and specific β-catenin inactivation lead to alterations of proliferation, cell cycle and apoptosis which lead to a dramatic decrease in tumor development in a tumor model for ACC. Further experiments are needed in order to know if β-catenin inactivation suppresses the growth or prevents engraftment of the cells. Nevertheless, these results confirm 1/the biological consequence of Wnt/β-catenin pathway activation in adrenocortical tumorigenesis, and 2/the major therapeutic interest to target this pathway.

Inhibition of Wnt/β-catenin pathway in a subgroup of aggressive ACC seems to be a interesting therapeutic target and should be evaluated in more detail in the future.

## Supporting Information

Table S1 PCR conditions and antibodies used.
(XLS)
